# Effect of season on physiological, biochemical, hormonal, and oxidative stress parameters of indigenous sheep

**DOI:** 10.14202/vetworld.2017.650-654

**Published:** 2017-06-16

**Authors:** Sawankumar D. Rathwa, A. A. Vasava, M. M. Pathan, S. P. Madhira, Y. G. Patel, A. M. Pande

**Affiliations:** 1 Department of Veterinary Physiology and Biochemistry, College of Veterinary Science and Animal Husbandry, Anand Agricultural University, Anand, Gujarat, India; 2 Department of Livestock Production Management, College of Veterinary Science and Animal Husbandry, Anand Agricultural University, Anand, Gujarat, India

**Keywords:** biochemical, indigenous sheep, physiological, temperature humidity index

## Abstract

**Aim:**

This study was conducted to evaluate the impact of summer and winter season on physiological, biochemical, hormonal, and antioxidant parameters in Indigenous sheep.

**Materials and Methods:**

The research was carried out during summer and winter season. 8 adult apparently healthy female sheep (aged 2-4 years) of similar physiological status were selected. Daily ambient temperature and relative humidity were recorded to calculate the temperature-humidity index (THI). The THI value of summer and winter season were 82.55 and 59.36, respectively, which indicate extreme hot condition during summer season and extreme cold condition during winter season. Physiological parameters were recorded daily during the experimental periods. Blood samples were collected at weekly interval and analyzed for biochemical, hormonal, and antioxidant parameters. The results were analyzed using completely randomized design.

**Results:**

From data obtained in this study, we found that higher THI during summer have significant effect over various physiological, biochemical, hormonal, and enzymatic indices of indigenous sheep. The physiological response such as rectal temperature, respiration rate (RR), pulse rate (PR), and skin temperature (ST) was increased significantly. We also found a significant increase in some biochemical parameters such as blood urea nitrogen (BUN), uric acid, creatinine (Cr), alanine transaminase (ALT), aspartate transaminase (AST), sodium (Na), and potassium (K). The level of cortisol hormone and superoxide dismutase (SOD), glutathione peroxidase (GPx), and lipid peroxidase (LPO) antioxidants increased significantly during summer. Whereas, some parameters such as glucose, cholesterol, calcium (Ca), inorganic phosphorus (IP), triiodothyronine (T_3_), and thyroxine (T_4_) were decreased significantly during summer season.

**Conclusion:**

It was concluded that the THI is a sensitive indicator of heat stress and is impacted by ambient temperature more than the relative humidity in Indigenous sheep. Higher THI is associated with significant increase in RT, RR, PR, ST, BUN, uric acid, Cr, ALT, AST, Na, K, cortisol, SOD, GPx, and LPO and with a significant decrease in glucose, cholesterol, Ca, IP, T_3_ and T_4_.

## Introduction

India is a mega diverse nation, housing around 10% of world’s species. Sheep is an important livestock species of India contributing greatly to food, fiber, rural employment, and gross domestic product. Sheep with its multifaceted utility (for meat, wool, skin, manure, and to some extent milk) plays an important role in the Indian agrarian economy. The variation in climatic variables such as temperature, humidity, and radiations are recognized as the potential hazards in the growth and production of all domestic livestock species. High ambient temperature accompanied by high air humidity and low ambient temperature cause an additional discomfort and enhances the stress level which in turn resulted in depression of the physiological and metabolic activities of these animals. Heat stress has been of major concern in reducing animal’s productivity in tropical, subtropical, and arid areas among the stressors [1]. Sweating, high respiration rate (RR), vasodilation with increased blood flow to skin surface, high rectal temperature, reduced metabolic rate, decreased dry matter intake, efficiency of feed utilization, and altered water metabolism are the physiological responses those are associated with negative impacts of heat stress on production and reproduction in sheep [2]. These physiological adjustments are essential to maintain normal body temperature and prevent hyperthermia [3-5]. Moreover, under these conditions, the animal’s productivity gets severely affected that result in a tremendous economic loss for the sheep industry in India.

Hematochemical tests are important tools for evaluation of physiological and health status of farm animals and almost indispensable in organic farming, where allowed veterinary interventions are strictly regulated and limited in scope. Hematological, biochemical, and oxidative stress analyses in farm animals have been extensively discussed as an essential part of clinical examination often pointing to a specific differential diagnosis or suggesting a prognosis. It is well recognized that physiological and hematochemical parameters are influenced by several factors including breed, age, reproductive status, housing, starvation, environmental factors, stress, and transportation [6-10]. These differences suggest the need to establish appropriate physiological baseline values for livestock which could be used in the realistic evaluation of the management practice, nutrition and diagnosis of health condition as well as in determining the physiological status of animals.

In view of the above-mentioned fact, the present investigation was planned on indigenous sheep to develop the database for alterations in various physiological, biochemical, hormonal, and oxidative stress parameters during summer and winter seasons for the development of sheep industry in Anand.

## Materials and Methods

### Ethical approval

The research was approved by the Institutional Animal Ethics Committee (IAEC, No.: 201/VPY/2015).

### Experimental site

The whole experiment was conducted during summer (15/04/2015 to 14/06/2015) and winter (01/12/1015 to 31/01/2016) seasons at Department of Veterinary Physiology and Biochemistry, College of Veterinary Science and AH, Anand Agricultural University, Anand. Anand town is situated in Middle Gujarat at 22°: 33’ North latitude and 72°: 57’ East longitude at an elevation of 39 m above the mean sea level with subtropical climate. On the basis of last 50 years (1956-2011) observation, the climatic condition of Anand is cold and dry (Av. Temp is 27.81°C-32.40°C) during winter (mid-October to mid-February) and hot and dry (Av. Temp is 34.64°C-40.08°C) during summer season (mid-February to mid-June).

### Experimental animals and feeding

About 8 adults (aged 2-4 years) apparently healthy indigenous sheep were selected from the flock maintained at Instructional Livestock Farm Complex, Department of Livestock Production Management, College of Veterinary Science and AH, Anand Agricultural University, Anand. The experimental animals were maintained on mixture of greens and dry roughages @1 kg/animal/day and concentrate @ 300 g/animal/day which was in accordance with ICAR 1998 feeding standards [11].

### Meteorological observation

Data of temperature and humidity of the shed in which animals were kept were recorded daily with the help of sling psychrometer during the period of study. This was used for calculation of temperature humidity index (THI) (Table-1). THI was calculated using the formula of Mader *et al*. 2006 [12].

**Table-1:** Temperature, relative humidity and THI of the summer and winter season.

Season	Ambient temperature (°C)	RH (%)	THI
Summer	33.72±1.52	54.88±1.52	82.55±1.52
Winter	20.73±2.24	62±2.24	59.36±2.24

RH=Relative humidity, THI=Temperature humidity index

### Physiological parameters

Physiological parameters, *viz*., RR (breath/min), pulse rate (PR) (rate/min), RT (°F), and skin temperature (ST) (°F) were measured by flank movement, palpation of femoral artery, clinical thermometer and infrared thermometer, respectively during the experimental period.

### Biochemical parameters

Blood samples were collected in heparinized and serum clot activator vacutainers (BD Vacutainer®, India) aseptically from the jugular vein of the experimental animals at weekly interval during summer (15/04/2015-14/06/2015) and winter (01/12/1015-31/01/2016) seasons. Serum was separated by centrifugation at 3000 rpm for 10 min and stored at −20°C till analyzed, whereas plasma was separated from the sample collected in heparinized vacutainers by centrifugation at 3000 rpm for 10 min and stored at −20°C and used for estimation of antioxidant parameters such as superoxide dismutase (SOD) [13], lipid peroxidase (LPO) in term of malondialdehyde production [14], and glutathione peroxidase (GPx) [15]. Serum biochemical parameters such as metabolites such as glucose, blood urea nitrogen (BUN), total protein (TP), uric acid, creatinine (Cr), cholesterol, minerals such as sodium (Na), potassium (K), calcium (Ca), inorganic phosphorus (IP), enzymes such as alanine transaminase (ALT), aspartate transaminase (AST), and hormones such as triiodothyronine (T_3_), thyroxine (T_4_), and cortisol were estimated. The serum metabolites, enzymes, and minerals such as Ca and IP were estimated using “Coral” kits (Coral®, India) in the chemistry analyzer (MINDRAY BS- 120 Chemistry Analyzer). The serum Na and K were estimated using flame photometer 128 (SYSTRONICS). The serum T_3_, T_4_ and cortisol were estimated by radioimmunoassay techniques using assay kits manufactured by Immunotech®, Beckman Coulter, Czech Republic. The laboratory analysis of the blood, plasma and serum was performed on the day of blood collection.

### Statistical analysis

Statistical analysis was performed using completely randomized design through SPSS Software. The model used for analysis was Y_ij_= μ+S_i_+E_ij_ where, Y_ij_ was observation of dependent variable; μ was the population mean for the variable; S_i_ was effect of season and E_ij_ was the random error associated with observation [16].

## Results

Based on data of ambient temperature and relative humidity, the calculated THI were 82.55 during summer and 59.36 during the winter season (Table-1). All the physiological parameters such as RT, RR, PR, and ST were significantly (p<0.05) higher during summer season as compared to winter season (Table-2). Among all biochemical parameters, the level of some parameters such as serum BUN, uric acid, Cr, AST, ALT, Na, and K was increased significantly (p<0.05), whereas rest of all was decreased significantly (p<0.05) during summer season (Table-3). The serum hormone concentration of T_3_ and T_4_ were 1.75 and 1.25 times decreased (p<0.05) while the concentration of cortisol was 1.29 times increased (p<0.05) during the summer season as compared to winter season (Table-3). The plasma oxidative stress parameters such as SOD, LPO, and GPx were 1.46, 1.31, and 1.29 times higher (p<0.05) during summer season than the winter season, respectively (Table-4).

**Table-2:** Physiological parameters (mean±SE) during summer and winter season.

Parameters	Summer	Winter
RT (°F)	102.17±0.04*	99.96±0.03
RR (breath/min)	40.02±0.5*	33.66±0.4
PR (rate/min)	81.8±0.5*	78.64±0.4
ST (°F)	100.1±0.02*	95.93±0.04

* p<0.05 (significant between column), SE=Standard error, RT=Rectal temperature, RR=Respiration rate, PR=Pulse rate, ST=skin temperature

**Table-3:** Biochemical parameters (mean±SE) during summer and winter season.

Parameters	Summer	Winter
Glucose (mg/dl)	46.15±1.30*	55.55±1.19
BUN (mg/dl)	22.87±0.85*	14.09±0.90
TP (g/dl)	6.78±0.19	6.70±0.25
Cr (mg/dl)	2.33±0.04*	0.98±0.04
Uric acid (mg/dl)	0.40±0.03*	0.18±0.01
Cholesterol (mg/dl)	44.29±0.43*	81.62±1.77
ALT (U/L)	44.51±1.53*	36.32±1.20
AST (U/L)	121.38±2.48*	103.86±3.27
Na (mmol/l)	161.24±0.37*	143.17±0.67
K (mmol/l)	6.00±0.05*	4.60±0.05
Ca (mg/dl)	7.49±0.11*	8.52±0.23
IP (mg/dl)	3.74±0.09*	5.70±0.49
T3 (ng/ml)	1.46±0.09*	2.55±0.12
T4 (ng/ml)	43.09±1.25*	54.93±1.05
Cortisol (ng/ml)	13.71±0.36*	10.62±0.25

* p<0.05 (significant between column), SE=Standard error, BUN=Blood urea nitrogen, TP=Total protein, Cr=Creatinine, ALT=Alanine transaminase, AST=Aspartate transaminase, Na=Sodium, K=Potassium, Ca=Calcium, IP=Inorganic phosphorus, T3=Triiodothyronine, T4=Thyroxine

**Table-4:** Oxidative stress parameters (mean±SE) during summer and winter season.

Parameters	Summer	Winter
SOD (U)	6.56±0.02*	4.49±0.02
LPO (nmol of MDA/ml of packed cells)	4.54±0.02*	3.46±0.02
GPx (U/ml)	98.45±0.11*	75.89±1.22

* p<0.05 (significant between column), SE=Standard error, SOD=Superoxide dismutase, LPO=Lipid peroxidase, GPx=Glutathione peroxidase, MDA=Malondialdehyde

## Discussion

Relative humidity was very high during winter season than summer season, whereas ambient temperature was very high during summer season as compare to winter season. THI was higher during the summer season. Thus, it was considered that the higher ambient temperature causes higher THI during summer season and makes the summer more stressful as compared to winter.

Increased RT is considered to be a good indicator of heat stress in animals [17]. Significantly increased RT with increase in THI during summer season indicates that the animals were in heat stressed condition [18]. During the summer season, higher RR may be due to adaptive mechanism of heat loss [19] and increased PR and ST may be due to vasodilatation of skin capillary bed and consequently increase in blood flow to body surface areas to facilitate heat dissipation [20].

We found significantly (p<0.05) increased level of BUN, uric acid and Cr during summer season. The increased level of these may be due to reduced blood flow toward kidney during heat stress condition. Similar findings of increased BUN, uric acid, and Cr during summer season have been reported by Srikandakumar *et al*. [17], Suhair [21], Ghosh *et al*. [22], and Indu *et al*. [23]. The decreased level of glucose [18,23-27] and cholesterol [23-26] during summer season may be due to decreased in feed intake. The increased level of serum ALT and AST was observed during the summer season. Increased in ALT and AST in hot period is in agreement with the findings of Srikandakumar *et al*. [17], Banerjee *et al*. [18], Wojtas *et al*. [20], Sharma and Kataria [28], and Al-Haidary *et al*. [29]. The increase in ALT and AST may be due to increase in gluconeogenesis or due to some deleterious effect of heat stress may occurred on liver activity [18,20].

The increased serum Na and K concentrations observed during the study may be due to dehydration. However, it also depending on the amount of water intake, which was not measured in the present study but sheep had *ad libitum* access to water [20,29]. The lower concentrations of Ca and IP observed during summer season may be due to decreased feed intake [26,29,30].

The lower concentration of T_3_ and T_4_ observed during the summer season that may be due to direct effect of heat stress on thyroid gland activity as well as due to reduced feed intake to avoid extra metabolic heat load [18,23,25,31]. We found increased level of cortisol during hot summer. Cortisol is well known as stress marker [20]. Higher temperature and THI during summer cause heat stress and in response to this the level of cortisol may increase [23,31]. Heat stress is also responsible for increased production of free radicals, which cause oxidative stress [22]. The SOD, LPO and GPx are well-known markers of oxidative stress [32]. The higher concentration of SOD, LPO, and GPx observed during summer season may be due to the heat stress conditions [22,33,34].

## Conclusion

It was concluded that the THI is a sensitive indicator of heat stress and is impacted by ambient temperature more than the relative humidity in indigenous sheep. Higher THI is associated with significantly increased level of RT, RR, PR, ST, BUN, uric acid, Cr, ALT, AST, Na, K, cortisol, SOD, GPx, and LPO and with significantly decreased level of glucose, cholesterol, Ca, IP, T_3_ and T_4_. These differences may be due to the direct or indirect effect of heat stress, dehydration and altered feeding pattern which necessitates further studies to ascertain precisely.

## Author’s Contributions

This study was a part of SDR’s original research work during M.V.Sc thesis programed. AMP has designed the plan of work. AAV helped during sample collection and laboratory analysis. MMP and SPM helped in statistical analysis and manuscript preparation. YGP provided animals and farm equipment. All the authors read and approved the final manuscript.
